# Very rare congenital malformation: single seminal vesicle, associated with unilateral Kidney Agenesis: first case report

**Published:** 2008-04-15

**Authors:** Mischianu Dan, Petre Ilie Cristian, Pacu Ovidiu, Rusu Florin

**Affiliations:** * Urology Clinic – University of Medicine and Pharmacy “Carol Davila”, Bucharest, Romania

**Keywords:** partial vesiculectomy, pelvic pain, renal agenesis, seminal vesicle

## Abstract

Introduction: The authors present the first case of a patient with single cystic seminal vesicle accompanied by left kidney agenesis, referring for diagnosis and treatment.

Material and methods: This is the case of a 22 year old patient presented for chronic pelvic pain. Investigations: abdominal and transrectal ultrasound as well as the computer tomography showed a cystic pelvic tumor with a diameter of around 8,5/6cm, and left kidney agenesis. The treatment for this condition varies from watchful waiting for asymptomatic cysts, to excision. We performed a plasty of the unique seminal vesicle.

Results: The postoperative evolution was uneventful with pain remission and improvement of ejaculatory function.

Conclusions: Single seminal vesicle has a very low incidence. For this pathology, partial vesiculectomy represents a viable therapeutic option in order to preserve sexual function in the case of a young patient.

## Introduction

The association between a seminal vesicle cyst and renal agenesis is an extremely rare lesion appearing in around 0.0021428% [**1**]. 

This is the first report of a malformation consisting of single seminal vesicle and renal agenesis. 

Genital and urinary systems are embryologically connected [**1**, **2**]. 

The ejaculatory ducts develop from the Wolffian duct system,. Just proximal of the ejaculatory duct, from the mesonephric duct, the seminal vesicles develop as a blind diverticulum at the age of approximately 12 fetal weeks. [**3**] 

The majority of patients with seminal cysts remain asymptomatic. The symptomatic ones require surgical management. Open surgery has been considered the definitive form of treatment with excellent results [**4**]. Recently, there have been some cases reported of laparoscopic management [**5**]. 

## Materials and methods:

**Fig. 1:**
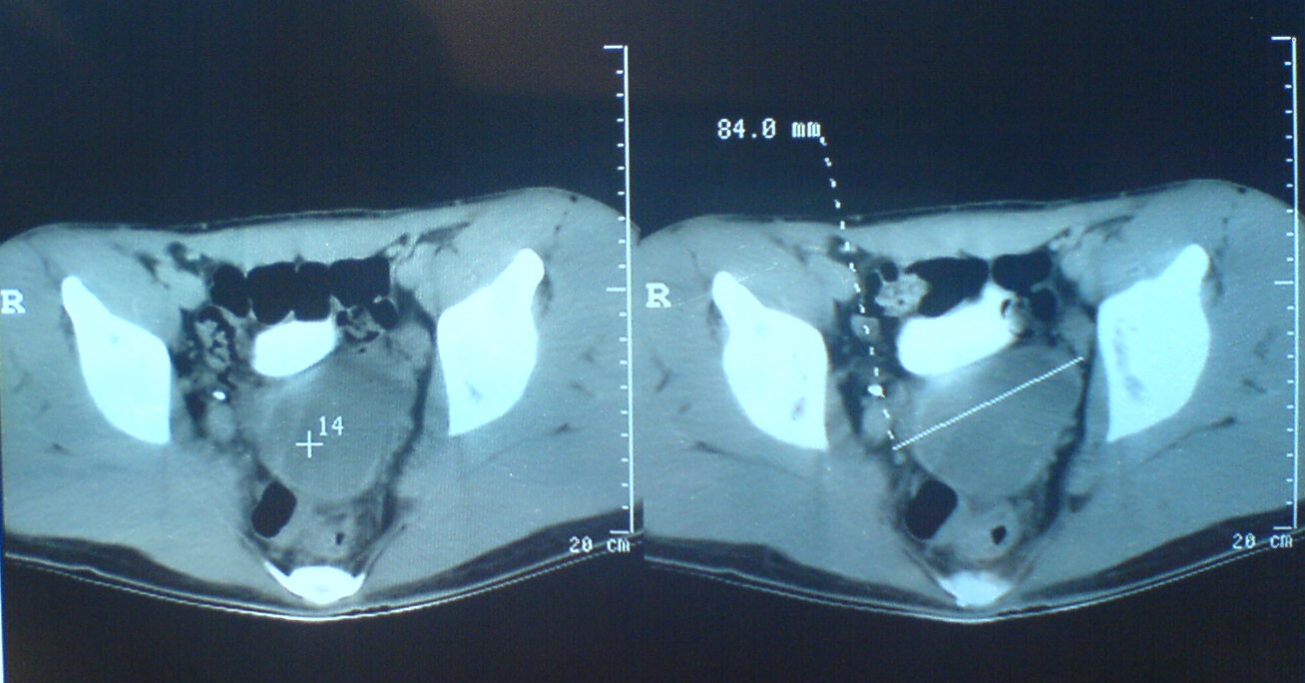
CT is showing the presence of a non enhancing cystic mass in the pelvic region, with a diameter of 84mm.

A 22- year-old male with history of hypogastric and ejaculatory pain was referred to our department after noticing at an enhancing CT (computer tomography) scan, the absence of the left kidney and the presence of a non enhancing cystic mass, with a diameter of around 84mm, situated in the pelvic region. (**Fig. 1**) He had no haematuria or spermaturia. 

Clinical examination revealed a mild firmness between the rectum and the bladder on bimanual palpation. The ultrasound examination could not identify the left kidney and measured a seminal vesicle of 61.8mm/ 41.5mm in sagittal section. The cyst was also evaluated with trans-rectal ultrasound. The intravenous urography could not identify the left kidney. The right kidney presented no abnormality. The cistoscopy visualized only the right uretheral orifice.

We concluded on a diagnosis of congenital malformation consisting in left renal agenesis and left cystic seminal vesicle. Thanks to the good results of the surgical management in the alleviation of symptoms, we recommended a classic approach. Preoperatively, we performed a catheterization of the right urether with a JJ stent in order to identify and protect it more easily during intervention. 

**Fig. 2:**
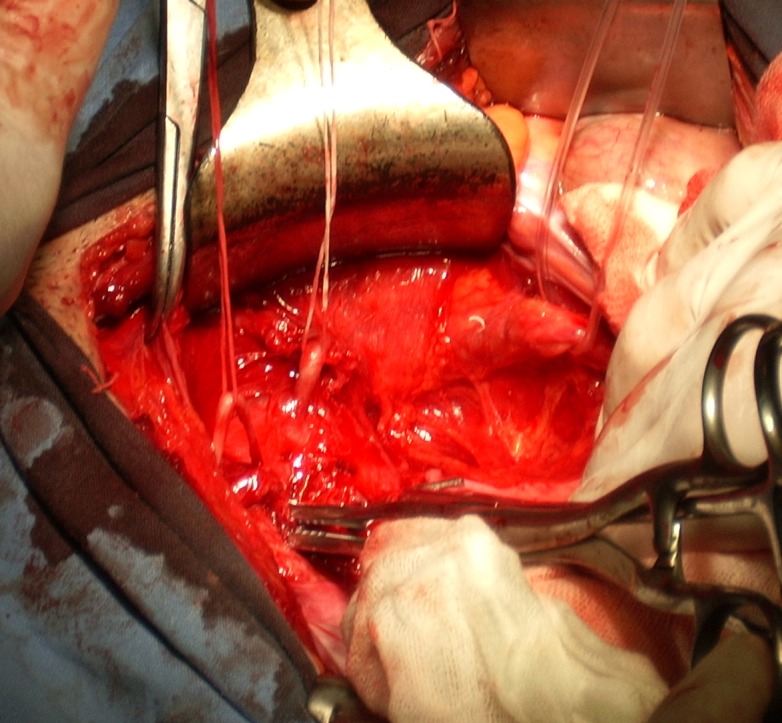
Intraoperatively: deferent ducts are dissected and isolated, both of them leading to one cystic seminal vesicle.

**Fig. 3:**
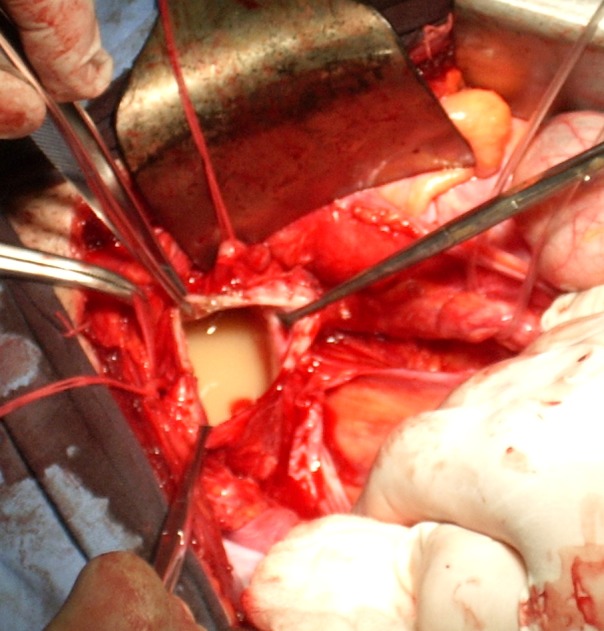
Intraoperatively: the seminal vesicle is filled with a thick whitish liquid.

Intraoperatively, the deferent ducts were dissected and isolated, both of them leading to one cystic seminal vesicle (**Fig. 2**). Because of the patient’s age (22) and in order to avoid interference with his fertility, we decided that instead of the left cystic seminal vesicle excision, as we planned preoperatively, we would perform a partial excision of the seminal vesicle with the preservation of the openings of both deferent ducts. When the cyst was incised, a thick, whitish fluid was evacuated and we were able to verify the permeability of the deferent ducts lumen on both sides (**Fig. 3**).

**Fig. 4:**
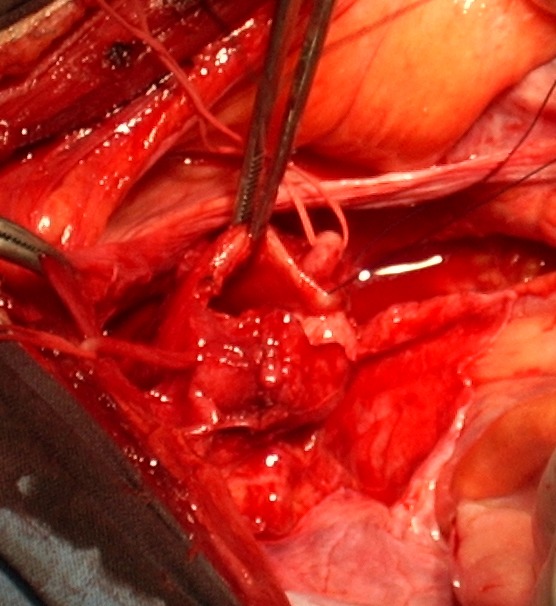
Intraoperatively: after excising a portion of the seminal vesicle, this is sutured with absorbable polyglactin 3.0.

After excising a portion of the vesicle, we sutured with absorbable polyglactin 3.0 (**Fig. 4**).

## Results:

Postoperative evolution was uneventful. 

**Fig. 5:**
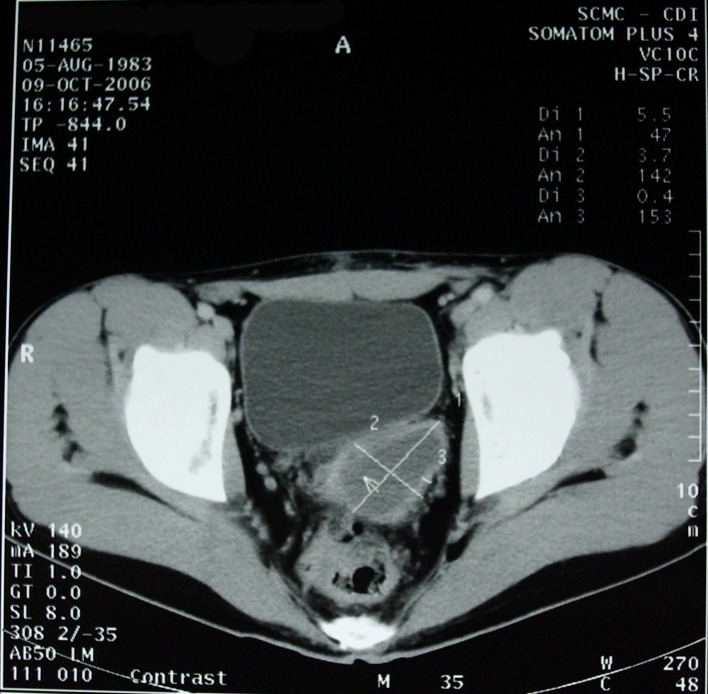
Postoperative CT scan, showing the seminal vesicle.

During postoperative follow-up, CT scan identifies the seminal vesicle with a diameter of 3.7/5.5cm. (**Fig. 5**)

One year after intervention, the patient is asymptomatic. The ultrasound examination measures a cystic seminal vesicle of 39/24 mm. 

## Conclusions:

This is the first report of a patient with single seminal vesicle associated with left renal agenesis. For the case of a single cystic seminal vesicle the diminution plasty represents a viable therapeutic option in order to preserve sexual function in the case of a young patient. 
